# Understanding Channel Complementarity for Multiplex Cell Segmentation

**DOI:** 10.1145/3807503.3819460

**Published:** 2026-07-28

**Authors:** Haotian Ma, Yi Lin, Elizabeth Godschall, Bruno Matuck, Kevin Matthew Byrd, Yifan Peng, Jinze Liu

**Affiliations:** Weill Cornell Medical College, New York, NY, USA; Virginia Commonwealth University, Richmond, VA, USA; Weill Cornell Medical College, New York, NY, USA; Virginia Commonwealth University, Richmond, VA, USA; Hospital Israelita Albert Einstein, São Paulo, Brazil; Virginia Commonwealth University, Richmond, VA, USA; Weill Cornell Medical College, New York, NY, USA; Virginia Commonwealth University, Richmond, VA, USA

**Keywords:** cell segmentation, Cellpose, multi-channel imaging, Applied computing → Imaging, Computing methodologies → Biometrics

## Abstract

Accurate cell segmentation in high-plex imaging remains challenging because no single biomarker channel captures the full structural diversity of cells, and many practical segmentation backbones are trained on a small, fixed-channel input convention. In this work, we study channel complementarity under the standard three-channel input convention of Cellpose-SAM using a systematic fused-evaluation framework with combination-specific operating-point calibration. On a primary healthy salivary gland region, we show that multi-channel gains are selective but meaningful: the strongest pair improves over the single-channel baseline, and the strongest triplet further improves over the best pair. On a second held-out diseased tissue region, we identify strong auxiliary combinations centered on EPCAM, and the triplet combination (DAPI, EPCAM, CD31) remains beneficial across both regions. To test whether these gains are artifacts of conservative post-processing, we additionally perform controlled-merge sensitivity analysis across the merge hyperparameter grid. These analyses do not reproduce the fused triplet gains, suggesting that the observed improvements arise from joint multi-channel inference rather than from the merge heuristic itself. Together, our results show that higher-order channel complementarity is real but selective under the standard three-channel input convention of Cellpose-SAM, and that reproducible triplet benefits can be identified through systematic search.

## 1 Introduction

**Figure 1:**
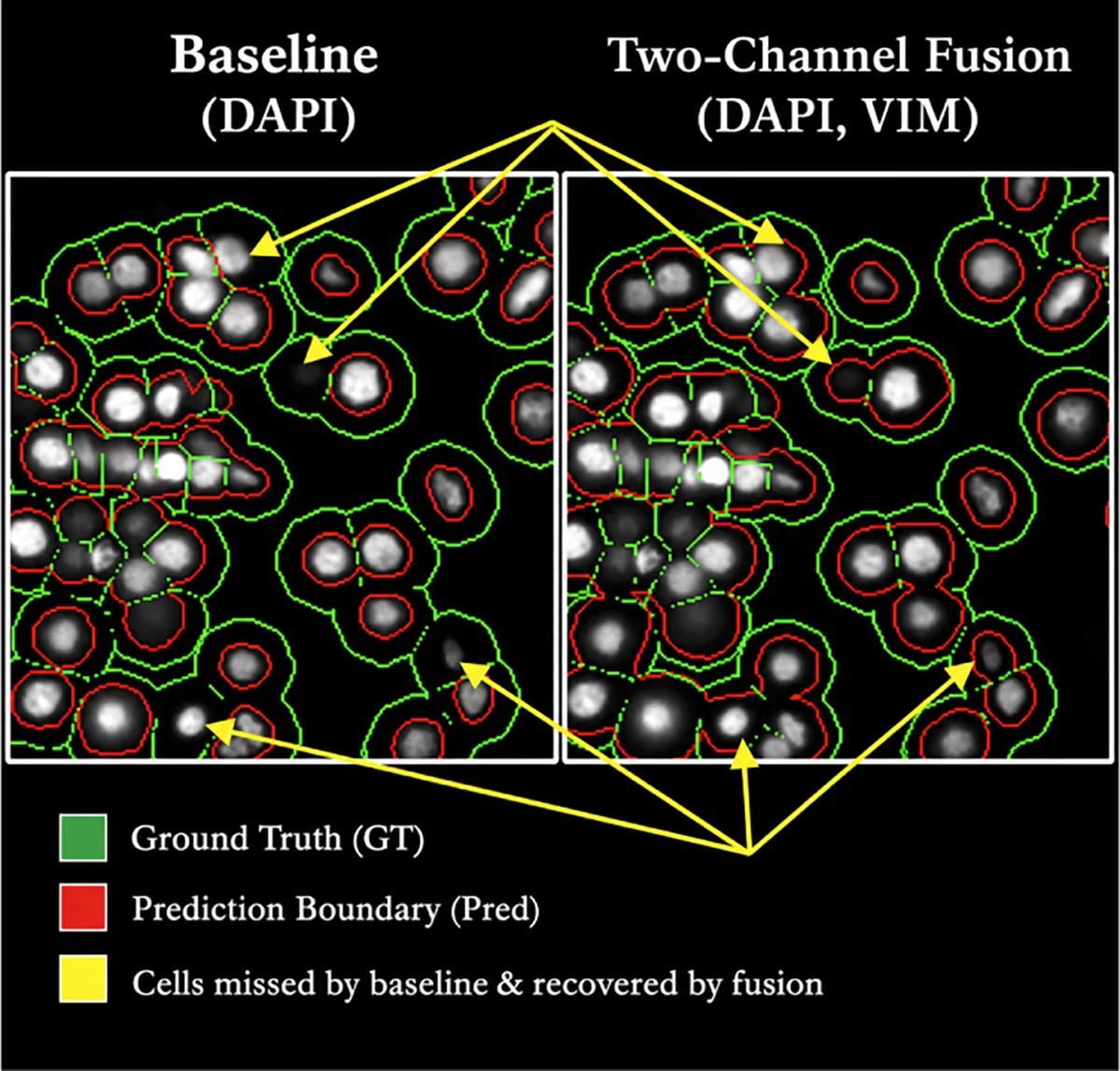
Multi-channel biomarker fusion can recover cell instances that are missed by single-channel segmentation. The baseline result (left) uses DAPI only, while the fused result (right) combines DAPI with VIM. Ground-truth cell boundaries are shown in green, and predicted boundaries are shown in red.

High-plex fluorescence-based imaging has substantially increased the dimensionality of cellular imaging, enabling the simultaneous observation of dozens of protein markers within a single tissue specimen. Such multiplexed measurements are a central component of modern spatial biology [11, 12], where understanding the spatial organization of cells and their molecular states is essential for studying tissue architecture, immune responses, and disease progression. While these technologies provide rich structural and molecular information, they also introduce new computational challenges for downstream image analysis tasks such as cell segmentation. As illustrated in Figure 1, segmentation based on a single structural channel may fail to detect certain cell instances that become identifiable when complementary channels are incorporated.

Many widely used segmentation architectures [19], including Cellpose-based models, operate with one to three channels. As a result, segmentation pipelines often rely on a single structural channel (e.g., a nuclear stain) while ignoring potentially informative signals in additional channels. However, many auxiliary channels contain complementary information that can clarify cell boundaries, highlight specific cellular compartments, or reveal structures that are weakly expressed in the baseline channel [8, 22]. Therefore, incorporating such complementary signals has the potential to improve segmentation quality.

In this work, we study biomarker complementarity under the standard three-channel input convention used by Cellpose-SAM [16], without modifying the model architecture or pretrained weights. Specifically, we use a systematic fused-evaluation framework with combination-specific operating-point calibration to compare baseline, pairwise, and triplet channel combinations under a consistent inference pipeline. Our goal is a controlled empirical analysis of how different channel combinations affect segmentation quality under a fixed pretrained backbone, including which auxiliary biomarkers provide meaningful segmentation benefit, whether higher-order triplet effects exist beyond strong pairs, and whether such effects remain observable across independent tissue regions.

## 2 Related Work

Cellpose [17, 20, 21] introduced a flow-field-based formulation for instance segmentation that has been widely adopted for cell and nucleus segmentation [2, 7] across imaging modalities [3, 8, 10, 15, 23]. More recently, Cellpose-SAM combines transformer-based feature extraction with the Cellpose segmentation paradigm to improve robustness to irregular cell morphology and scale variation. However, many Cellpose-based pipelines remain constrained to one to three input channels due to architectural design and pretrained-weight compatibility. This limitation poses challenges for high-plex imaging datasets, where dozens of channels may be available.

Multiplex fluorescence microscopy [5, 6, 18] provides rich structural information [9], but integrating multiple channels into segmentation workflows raises several challenges: identifying complementary channels, combining signals without introducing false positives, and determining whether additional channels provide meaningful benefit. Existing approaches [1, 4, 13, 14, 22] typically rely on early fusion through channel concatenation, late fusion of independently predicted masks, or learned fusion modules. However, naive late-fusion strategies, such as mask unions, often propagate artifacts from individual channels, inflating false positives. In contrast, our study evaluates channel combinations under the practical fixed-input pretraining convention of Cellpose-SAM, using a controlled operating-point-normalized protocol to assess the marginal value of additional channels.

## 3 Materials and Methods

### 3.1 Multiplex Imaging Dataset

The experimental dataset consists of two human major salivary gland regions (See details in Section A.1 of the Supplementary Material): a Healthy region from a normal salivary gland and a Diseased region from a salivary gland affected by graft-versus-host disease (GVHD). Ground-truth cellular boundaries were established using a human-in-the-loop annotation workflow supervised by an expert oral pathologist.

Table 1 summarizes the dataset statistics used in this study. For the Healthy region, the annotated region is partitioned into fixed 1, 000 × 1, 000 pixel patches, from which 50 are randomly sampled to form a development set used for operating-point calibration, and 10 non-overlapping patches are sampled as an independent test set for performance evaluation. The Diseased region is used only as an independent held-out test region with 10 fixed-size test patches for cross-region validation. Supplementary Table S1 summarizes the representative channels discussed later in the channel-complementarity analysis, including the baseline nuclear channel and the top-performing complementary biomarkers identified in the repeated experiment.

### 3.2 Cellpose-SAM

In this study, we use Cellpose-SAM [16] as a fixed backbone. To enable systematic comparison, we adapt each channel combination to the required three-channel format. For single-channel analysis, a selected channel is replicated across all three input slots, yielding an input of the form (*I*_*i*_, *I*_*i*_, *I*_*i*_). For two-channel analysis, we use (*I*_0_, *I*_*i*_, *I*_0_) so that the baseline structural channel (DAPI) remains present while the auxiliary channel is introduced into the second slot. For three-channel analysis, we use (*I*_*0*_, *I*_*i*_, *I*_*j*_).

Two thresholds control Cellpose-SAM inference. The *flow threshold* controls how consistently predicted pixel flows must agree to form a valid instance mask. In contrast, the cell probability threshold determines how much cell evidence is required for a region to be accepted as part of a cell. Lower flow thresholds generally admit more candidate instances, which can improve recall but also increase spurious detections. The *cell probability threshold* allows proposals in weaker or more ambiguous regions. Because segmentation performance is sensitive to these parameters, threshold calibration is a key component of our evaluation pipeline.

### 3.3 Evaluation pipeline

**Figure 2:**
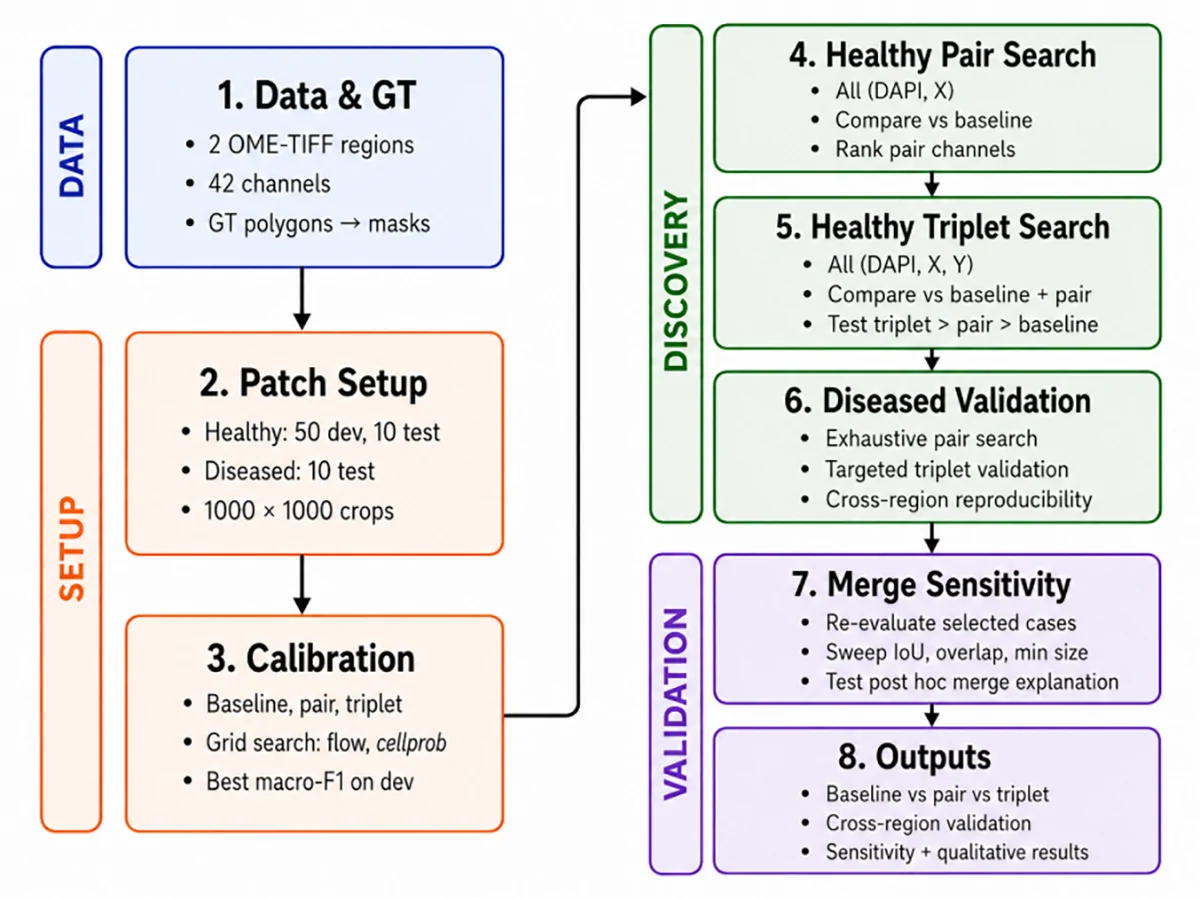
Evaluation workflow.

Figure 2 summarizes the evaluation workflow. For each evaluated input configuration, operating points are calibrated on the Healthy region development crops by grid search over the flow and cell probability thresholds. The selected thresholds are then applied to held-out test crops for operating-point-normalized comparison across channel combinations.

The primary analysis is performed on the Healthy region. Pairwise combinations of the form (DAPI, *X*) are evaluated exhaustively against the DAPI baseline, followed by exhaustive triplet evaluation over (DAPI, *X, Y*) combinations to determine whether higher-order channel fusion provides additional benefit beyond the strongest pair. The diseased region is then used for cross-region validation through exhaustive pair search and targeted triplet evaluation based on the strongest pairwise backbones.

Segmentation performance is measured using instance-level precision, recall, and F1 score. Predicted instances are matched to ground-truth instances using an intersection-over-union (IoU) threshold of 0.4. Metrics are computed per patch and macro-averaged to prevent patches with unusually high cell density from disproportionately influencing the overall score.

In addition to fused multi-channel inference, selected cases are re-examined using controlled-merge sensitivity analysis. These diagnostic experiments sweep IoU, overlap, and minimum-size thresholds to test whether the observed triplet gains can be reproduced by post hoc merging rather than by joint multi-channel inference itself.

## 4 Results

### 4.1 Main Quantitative Results

Table 2 summarizes the main quantitative results across the two evaluated regions.

#### Baseline behavior differs across the two evaluated regions.

On the Healthy region, the DAPI-only baseline achieves a macro-averaged precision of 0.619, a recall of 0.518, and an F1 score of 0.561. This pattern suggests that DAPI alone provides a usable but incomplete structural signal in this region, with a substantial fraction of true instances remaining undetected. In contrast, the baseline on the Diseased region is much stronger, reaching 0.854 precision, 0.747 recall, and 0.794 F1. Thus, the two regions do not start at the same baseline difficulty level. Healthy region presents a more challenging setting for DAPI-only segmentation, whereas Diseased region already supports relatively strong single-channel performance. Such behavior is consistent with the possibility that a single channel captures only part of the structural information needed for complete instance detection in this multiplex imaging setting, leading to incomplete instance detection [2, 8, 9].

#### Pairwise gains are selective, and EPCAM emerges as the strongest complementary channel.

On the Healthy region, the strongest pairwise result is obtained by combining DAPI with EPCAM, improving F1 from 0.561 to 0.574. Although this gain is modest in absolute magnitude, it establishes EPCAM as the best pairwise complement to the DAPI baseline after an exhaustive search in this region. On the Diseased region, the pairwise gains are larger overall. Again, the best pair is DAPI+EPCAM, which improves F1 from 0.794 to 0.846. Additional strong pairwise performance is also observed for DAPI+VIM and DAPI+CD68, with F1 values of 0.832 and 0.830, respectively. Taken together, these results show that pairwise gains are selective rather than universal, but that EPCAM is the most consistent complementary channel across the two evaluated regions.

#### Triplet evaluation revises the main conclusion on the Healthy region and shows selective higher-order gains on the Diseased region.

The exhaustive triplet search on the Healthy region identifies triplets that outperform both the baseline and the strongest pairwise result. In particular, the best triplet (DAPI, EPCAM, CD31) achieves an F1 of 0.620, exceeding both the DAPI baseline (0.561) and the strongest pair, DAPI+EPCAM (0.574). Additional high-ranking triplets, including (DAPI, EPCAM, VIM) and (DAPI, CD31, EPCAM), also remain above the pairwise baseline. Therefore, the revised ordering on the Healthy region is triplet > pair > baseline, rather than the pair-dominant pattern suggested by the earlier staged screen.

#### Cross-region validation supports reproducibility, but also highlights context dependence.

On the Diseased region, targeted triplet evaluation shows that positive triplet gains remain observable, although they are more selective than on the Healthy region. The triplet (DAPI, EPCAM, CD31) achieves an F1 of 0.849, improving over both the Diseased region baseline and the strong DAPI+EPCAM pair. A second positive case (DAPI, CD68, EPCAM) reaches an F1 of 0.837 and likewise exceeds the single-channel baseline. At the same time, the number of triplets that improve over their matched pairwise reference remains limited, indicating that higher-order channel complementarity is real but sparse. Overall, the cross-region results support two conclusions: first, the strongest triplet signal is not restricted to a single region, since (DAPI, EPCAM, CD31) is beneficial in both regions; second, the magnitude and frequency of triplet gains remain context-dependent across tissue regions.

### 4.2 Controlled-merge Sensitivity Analysis

Table 3 summarizes the controlled-merge sensitivity analysis. Here, we test whether the triplet gains observed in fused inference can be reproduced by post hoc mask-level merging of independently predicted single-channel instances under a grid of 27 merge settings.

For the comparison between (DAPI, EPCAM, CD31) and its matched pairwise reference (DAPI, EPCAM), the triplet-over-pair deltas are identically zero across all tested merge settings in both the Healthy and Diseased regions. This means that, under the controlled-merge formulation, adding CD31 does not recover any additional accepted instances beyond those already obtained from the matched pair. In contrast, for the comparison between (DAPI, CD68, EPCAM) and (DAPI, CD68) on the Diseased region, the triplet-over-pair deltas are uniformly negative across all 27 tested settings. Thus, the same merge-based procedure not only fails to reproduce the fused triplet improvement. But in this case, it performs consistently worse than the matched pairwise reference.

Taken together, these results do not support the interpretation that the main triplet gains reported in Table 2 arise from the controlled-merge heuristic. Instead, they are more consistent with benefits that emerge from joint multi-channel inference than from mask-level post-processing alone.

### 4.3 Qualitative Case Study

**Figure 3:**
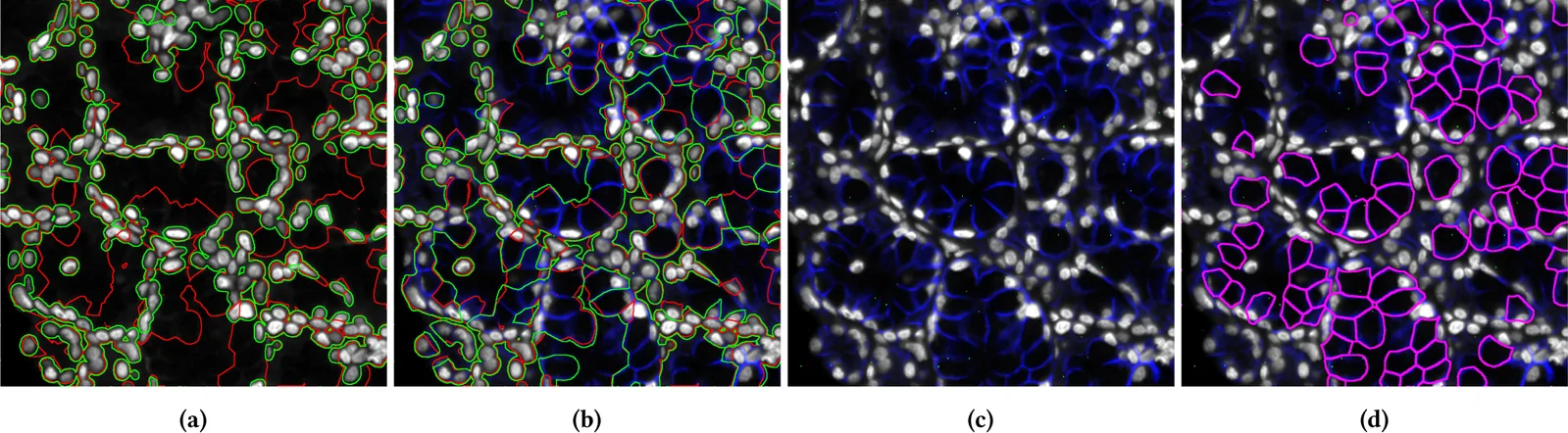
Qualitative comparison between the single-channel baseline and the selected multi-channel combinations on a representative held-out test patch. a, DAPI-only baseline (grayscale) with ground-truth (red) and predicted boundaries (green). b, Corresponding two-channel fusion using DAPI (grayscale) and EPCAM (blue) under the same evaluation framework. c, Spatial distribution of the triplet biomarkers DAPI (grayscale), EPCAM (blue), and CD31 (green). d, Instances detected by the triplet configuration (magenta) that are not recovered by the baseline, illustrating where the triplet provides additional instance-level benefit relative to DAPI alone.

Figure 3 provides representative qualitative examples illustrating the segmentation behavior of the baseline and selected fused configurations on a held-out test patch. The qualitative patterns are consistent with the quantitative results in Table 2: the DAPI-only baseline provides a reasonable structural starting point, pairwise fusion with EPCAM improves boundary support, and the triplet configuration (DAPI, EPCAM, CD31) recovers additional instances that are not captured by the baseline alone.

#### Baseline and pairwise comparison.

In the DAPI-only baseline (Figure 3a), many nuclei are localized clearly, but the resulting segmentation is still incomplete relative to the annotated cell population. Several ground-truth cell regions remain only partially covered or are not recovered as distinct predicted instances. Adding EPCAM (Figure 3b) provides additional boundary information around epithelial structures, producing a more complete pairwise segmentation while preserving the overall spatial organization visible in the baseline. This qualitative pattern is consistent with the strong pairwise performance of DAPI+EPCAM in both evaluated regions.

#### Triplet biomarker context.

Figure 3c shows the corresponding triplet biomarker composite, with DAPI in grayscale, EPCAM in blue, and CD31 in green. The three channels emphasize different aspects of local structure: DAPI highlights nuclear signal, EPCAM outlines epithelial-associated boundaries, and CD31 contributes additional context in surrounding vascular and stromal regions. This visual separation helps explain why the triplet configuration can provide information that is not fully captured by DAPI alone or by the strongest pairwise fusion.

#### Triplet-specific gains relative to baseline.

Figure 3d highlights instances detected by the triplet configuration but not recovered by the DAPI baseline. These triplet-specific detections cluster in localized regions where the single-channel baseline appears structurally ambiguous. This pattern supports the interpretation that the triplet gain arises from additional complementary context rather than from a uniform global shift in detection sensitivity. In conjunction with the quantitative results, the qualitative evidence supports the view that the triplet configuration can recover additional instances beyond the baseline and strong pairwise fusion on the Healthy region, while remaining consistent with the more selective, context-dependent triplet gains observed in cross-region validation.

## 5 Discussion

Our results show that under the standard three-channel input convention of Cellpose-SAM, the strongest triplet outperforms both the strongest pair and the single-channel baseline, yielding a triplet > pair > baseline ordering in the evaluated setting. At the same time, the gains are clearly selective rather than universal. Thus, the main message is not that adding more channels always improves segmentation, but that higher-order channel complementarity can be real and measurable when the added biomarkers contribute to an effective structural context.

Second, segmentation performance is shaped by tissue context rather than following a uniform improvement pattern. Across the two evaluated regions, the effect of channel fusion depends strongly on local tissue organization. DAPI provides a reliable nuclear anchor in both regions, but it lacks direct information about cell boundaries and higher-order tissue structure. As a result, its usefulness depends on how well the nuclear signal alone reflects the underlying architecture. On the Healthy region, where the tissue appears more regular and compartmentalized, DAPI combined with a single complementary marker, most notably EPCAM, is already sufficient to provide measurable improvement over the baseline. In contrast, Diseased region presents a more heterogeneous spatial environment, with stronger mixing across epithelial, immune, vascular, and stromal compartments. In this setting, the nuclear signal alone or simple pairwise fusion is often insufficient to fully resolve cell boundaries and local instance structure.

Third, the greatest improvements arise when added channels introduce complementary biological context. Among the tested biomarkers, EPCAM emerges as the most consistent complementary channel across both regions. This is biologically plausible in salivary gland tissue, where epithelial organization is a dominant structural component and epithelial boundaries provide information that is not fully recoverable from DAPI alone. In this setting, DAPI primarily localizes nuclei. In contrast, EPCAM provides an additional epithelial boundary-associated signal that helps separate adjacent cells and recover instances that remain ambiguous in the single-channel baseline. This interpretation is consistent with the robust performance of the DAPI+EPCAM pair on both regions.

Additionally, cross-region validation supports reproducibility, but also highlights context dependence. Exhaustive pair search on the Diseased region again identifies EPCAM as part of the strongest pairwise configuration, which supports its role as a robust complementary marker rather than a region-specific artifact. At the same time, the triplet gains on the Diseased region are more limited than on the Healthy region. Although (DAPI, EPCAM, CD31) remains beneficial and (DAPI, CD68, EPCAM) also improves over the baseline, the number of triplets that exceed their matched pairwise reference is small. This indicates that higher-order channel complementarity is directionally reproducible but variable in magnitude. This region dependence is expected in multiplex tissue imaging, where the usefulness of a biomarker depends not only on its molecular specificity but also on tissue architecture, cell-type composition, and the spatial arrangement presented to the model.

Finally, not all additional markers provide equal benefit. We observed that the utility of added channels is determined by both biological specificity and spatial redundancy. Markers such as VIM can be informative in pairwise settings, as observed on the Diseased region. Still, they may contribute less to higher-order combinations if their signal is spatially diffuse, broadly distributed, or partially redundant with information already supplied by stronger markers. More generally, the value of an added biomarker depends not only on whether it is biologically relevant, but on whether it introduces new spatial structure that the model can exploit under the chosen input convention. This helps explain why some biologically meaningful markers remain neutral or weak in triplet configurations, whereas others yield measurable gains.

This study has several limitations. First, the study is still based on a limited number of annotated regions, so the present conclusions should be interpreted as evidence of reproducible local behavior rather than as a definitive ranking of biomarkers for all salivary gland samples. Second, although exhaustive pair and triplet searches were feasible on the Healthy region, larger combination spaces would quickly become computationally expensive, especially if extended to additional tissues, regions, or segmentation backbones. Third, the conclusions are specific to the pretrained Cellpose-SAM setup and its standard three-channel convention; different model architectures or fine-tuned segmentation systems may exploit the same biomarkers differently. Fourth, our evaluation focuses on instance-level precision, recall, and F1 score; additional structural metrics, such as boundary displacement error, shape consistency, or topology-aware criteria, could provide further insight into segmentation quality. Finally, broader validation across distinct tissues, imaging platforms, and staining protocols will be necessary to determine how general these channel-complementarity patterns are beyond the present setting.

## 6 Conclusion

We present a systematic empirical characterization of channel complementarity in multiplex cell segmentation under the standard three-channel input convention used by Cellpose-SAM. Using an operating-point-normalized evaluation protocol, we compare baseline, pairwise, and triplet channel combinations while reducing confounding from threshold-selection effects. Results suggest that the value of additional biomarkers depends not only on their biological specificity, but also on tissue context and on how their information is jointly presented to the segmentation model.

## Supplementary Material

Supplemental Material

## Figures and Tables

**Table 1:** Statistics of the evaluated regions used in this study.

	Healthy Dev.	Healthy Test	Diseased Test

No. of patches	50	10	10
Patch size	1, 000 × 1, 000	1, 000 × 1, 000	1, 000 × 1, 000
Cell count	5,503	993	1,542
Cells per patch	110	99	154
Cell types	9	8	11

**Table 2:** Macro-averaged instance segmentation performance on the test set.

Channels	P	R	F1	ΔP	ΔR	ΔF1

Healthy region						
Baseline (DAPI)	0.619 (0.34)	0.518 (0.31)	0.561 (0.32)	–	–	–
(DAPI, EPCAM)	0.598 (0.30)	0.554 (0.33)	0.574 (0.33)	−0.021*	0.035**	0.013**
(DAPI, EPCAM, CD31)	0.635 (0.31)	0.615 (0.26)	0.620 (0.28)	0.016*	0.097**	0.059*
(DAPI, EPCAM, VIM)	0.716 (0.28)	0.609 (0.24)	0.612 (0.27)	0.097*	0.091**	0.051**
(DAPI, CD31, EPCAM)	0.631 (0.31)	0.591 (0.28)	0.608 (0.29)	0.012**	0.073**	0.047**
Diseased region						
Baseline (DAPI)	0.854 (0.03)	0.747 (0.1)	0.794 (0.07)	–	–	–
(DAPI, EPCAM)	0.884 (0.04)	0.811 (0.06)	0.846 (0.05)	0.030*	0.064***	0.052***
(DAPI, VIM)	0.855 (0.06)	0.810 (0.06)	0.832 (0.06)	0.001*	0.063***	0.038***
(DAPI, CD68)	0.891 (0.04)	0.784 (0.07)	0.830 (0.05)	0.037*	0.037**	0.036**
(DAPI, EPCAM, CD31)	0.888 (0.05)	0.813 (0.06)	0.849 (0.05)	0.034**	0.066**	0.055**
(DAPI, CD68, EPCAM)	0.893 (0.03)	0.792 (0.09)	0.837 (0.06)	0.039***	0.045*	0.043**

The baseline corresponds to DAPI replicated across the three input slots and evaluated at its globally optimal operating point (flow threshold = 0.5, cell probability threshold = −2) selected on the development set. Fusion combinations are assessed at their own optimized operating points. Metrics are reported as mean (std) across the test patches. Δ values denote (Fused – Baseline).

*: *p* < 0.1

**: *p* < 0.05

***: *p* < 0.01.

**Table 3:** Summary of controlled-merge sensitivity analysis.

Baseline pair	3rd channel	Δ*P*	Δ*R*	Δ*F*1	+	0	−

Healthy region							
(DAPI, EPCAM)	CD31	0.000	0.000	0.000	0	27	0
Diseased region							
(DAPI, EPCAM)	CD31	0.000	0.000	0.000	0	27	0
(DAPI, CD68)	EPCAM	−0.096	−0.089	−0.098	0	0	27

Triplet-over-pair deltas are evaluated over 27 merge settings. Default values correspond to the original merge setting (IoU = 0.6, overlap = 0.3, minimum size = 115).
