# Supplementary material for: Understanding Channel Complementarity for Multiplex Cell Segmentation
Source: ACM BCB. Author manuscript; Available in PMC 2026 Aug 27. (PMC13512904; doi:10.1145/3807503.3819460)
Supplement: Supplementary file 1 [file NIHMS2200229-supplement-Supplemental_Material.pdf]

A Supplementary materials

A.1 Multiplex Imaging Dataset

The experimental dataset consists of two human major salivary gland regions: a Healthy region from a normal salivary gland and a Diseased region from a salivary gland affected by graft-versus-host disease (GVHD). Both datasets were curated through the OMAPs classification of the HuBMAP project and comprise 42-antibody multiplexed fluorescence whole-slide images acquired using the Akoya PhenoCycler (CODEX) platform. This high-plex panel captures diverse cellular compartments and biological functions, including nuclear and structural markers (e.g., DAPI, Pan-Cytokeratin), immune lineage markers (e.g., CD20, CD3e), and functional or cell-state markers (e.g., PD-1), so that different channels may contribute complementary structural or molecular information for cell instance detection. The Healthy and Diseased regions correspond to two independent annotated multiplex image regions of size $13,751 \times 17,158$ and $17,109 \times 17,161$ pixels, respectively.

Ground-truth cellular boundaries were established using a human-in-the-loop annotation workflow supervised by an expert oral pathologist. An iterative active-learning strategy was employed to refine segmentation quality. In each iteration, the pathologist manually curated and corrected cell boundaries within selected regions of interest (ROIs), and the corrected annotations were used to retrain a Cellpose-based segmentation model. This process progressively improved boundary accuracy across complex tissue regions. The final annotations are provided as polygon-based instance masks and rasterized into pixel-level segmentation maps for evaluation.

A.2 Evaluation pipeline details

A.2.1 Operating Point Calibration. Segmentation performance is sensitive to the flow threshold and cell probability threshold, so we calibrate these parameters on the Healthy region development set and use the resulting operating-point-normalized framework for all subsequent evaluations.

For each evaluated baseline, pairwise, and triplet configuration on the Healthy region, we perform a threshold sweep over multiple pairs of flow and cell probability values on the development set. The threshold pair that maximizes the macro-averaged F1 score on the Healthy region development crops is selected as the optimized operating point for that configuration.

These selected thresholds are then applied to the held-out Healthy region test crops to evaluate primary discovery. The diseased region does not have a separate development split; instead, it is used only as an independent held-out test region for cross-region validation under the same evaluation framework.

A.2.2 Evaluation Framework Across Input Types. We consider three input types. For the single-channel baseline, the baseline structural channel is replicated across the three input slots. For pairwise analysis, we evaluate inputs of the form $stack(I_0, I_X, I_0)$, where I_0 denotes the baseline structural channel and I_X denotes one auxiliary channel. For triplet analysis, we evaluate inputs of the form $stack(I_0, I_X, I_Y)$, where two auxiliary channels are jointly presented with the baseline channel under the same three-channel input convention.

For each evaluated input type, segmentation performance is measured using instance-level precision, recall, and F1 score. Predicted instances are matched to ground-truth instances using an intersection-over-union (IoU) threshold of 0.4. Metrics are computed per patch and macro-averaged across patches to avoid domination by unusually dense regions.

A.2.3 Primary Discovery Experiment on the Healthy Region. Our primary discovery experiment is conducted on one annotated salivary gland region (Healthy region). We first establish a single-channel DAPI baseline using the calibration protocol described above. We then perform an exhaustive pair search over all candidate two-channel inputs (I_0, I_X, I_0), where I_X ranges over all non-baseline channels in the multiplex panel. Each pair is calibrated on the development set and evaluated on the test set.

To assess higher-order channel complementarity, we next perform an exhaustive triplet search over three-channel inputs (I_0, I_X, I_Y). As in the pair analysis, each triplet is assigned its own calibrated operating point on the development set and is then evaluated on the test patches. This exhaustive design allows us to determine whether specific triplets outperform both the single-channel baseline and the strongest pairwise combinations under the same evaluation framework.

The purpose of the Healthy region experiment is discovery rather than validation: it is used to identify the strongest baseline, pairwise, and triplet channel combinations under operating-point-normalized evaluation, with all operating-point calibration performed on the Healthy region development set.

A.2.4 Cross-region Validation on the Diseased region. To examine whether the main findings generalize beyond the discovery region, we conduct a second experiment on an independent held-out region (the Diseased region). Diseased region contains only 10 held-out test crops and is used exclusively for cross-region validation rather than for development-set calibration.

On this second region, we evaluate the DAPI baseline and perform an exhaustive pair search over all two-channel combinations on the held-out test crops. Because exhaustive triplet search is substantially more expensive, we use a targeted validation design for Diseased region. Specifically, we first identify the strongest pairwise combinations on the Diseased region, then evaluate targeted triplets formed by extending the top-pair backbones with additional third channels. This design allows us to test whether the strongest triplet patterns observed on Healthy region remain beneficial in an independent region while keeping the cross-region experiment computationally tractable.

We emphasize that the Diseased region experiment is a validation study rather than a second discovery sweep. It is intended to test whether strong fused combinations, especially those centered on the strongest pair backbones, remain competitive across regions.

A.2.5 Controlled-merge Sensitivity Analysis. In addition to the fused-inference experiments above, we perform a separate sensitivity analysis. This analysis is not used for primary discovery. Instead, it serves as a diagnostic test of whether the observed triplet gains can be reproduced by post hoc merging of independently predicted single-channel masks.

For this analysis, we first generate single-channel predictions for the channels involved in the selected pair and triplet configurations. We then apply a controlled merge procedure when integrating masks predicted from different channels.

Let $\{M_1, M_2, \ldots, M_K\}$ denote the set of instance masks predicted from the baseline structural channel, and let $\{C_1, C_2, \ldots, C_J\}$ denote candidate instance masks predicted from an auxiliary channel. For each candidate instance C_j, we compute the intersection-over-union (IoU) with each baseline instance M_i:

$$\mathrm{IoU}(C_j, M_i) = \frac{|C_j \cap M_i|}{|C_j \cup M_i|}. \tag{1}$$

A candidate instance is incorporated into the merged mask only if it satisfies all of the following criteria: (1) $\max_i \mathrm{IoU}(C_j, M_i) < 0.6$, (2) overlap ratio $\frac{|C_j \cap M_i|}{|C_j|} < 0.3$, and (3) $|C_j| \geq 115$ pixels. These thresholds were selected empirically to suppress small fragments and avoid incorporating instances that largely overlap existing baseline detections. In the sensitivity analysis, we sweep these merge hyperparameters across a predefined grid and compare the resulting merged masks against their corresponding pairwise baselines. This analysis allows us to test whether gains observed in fused triplet inference remain observable under conservative mask-level post-processing.

Table S1: Representative channels discussed in the channel-complementarity analysis.

Ch.	Biomarker	Description
0	DAPI	Nuclear stain used as the baseline structural channel.
7	EPCAM	Epithelial cell adhesion molecule; provides epithelial boundary-associated signal and is the most consistent complementary channel across the two evaluated regions.
19	CD68	Macrophage-associated marker; contributes to the immune-compartment context and supports a positive triplet result on the Diseased region when combined with DAPI and EPCAM.
22	CD31	Endothelial / vascular marker; contributes to the vascular context and forms the strongest reproducible triplet with DAPI and EPCAM.
37	VIM	Vimentin; stromal / mesenchymal-associated marker that remains strong in selected pairwise settings, particularly on the Diseased region.
